# Factors associated with general practitioners’ contacts with sick-listed patients’ employers: A Swedish nationwide questionnaire study

**DOI:** 10.1177/14034948211053141

**Published:** 2021-10-23

**Authors:** Paula Nordling, Kristina Alexanderson, Gunnel Hensing, Per Lytsy

**Affiliations:** 1 School of Public Health and Community Medicine, Sahlgrenska Academy, University of Gothenburg, Gothenburg, Sweden; 2 Primary Health Care, Region Västra Götaland, Sweden; 3 Division of Insurance medicine, Department of Clinical Neuroscience, Karolinska Institutet, Stockholm, Sweden

**Keywords:** Primary healthcare, primary care, family physician, general practitioner, sick leave, return to work, insurance medicine, professional practice, intersectoral collaboration

## Abstract

*Background:* General practitioners’ (GP) contacts with sick-listed patients’ employers have been shown to be of importance for return to work. This study aimed to explore GPs’ contacts with sick-listed patients’ employers and factors associated with such contacts. *Methods:* In this cross-sectional study, 4228 GPs responded to a nationwide questionnaire about sickness certification (SC) practices. Outcomes of interest were participation in stakeholder meetings, having other contacts with employers, and satisfaction with employer contacts. Logistic regression models were used to investigate associations with factors related to the GP and the GP’s workplace. *Results:* Among GPs, 34.8% participated in stakeholder meetings and 15.1% had other employer contacts; 39.4% had any or both of these contacts. Of GPs who had contacts with patients’ employers, 65.8% were satisfied with the contacts. GPs regularly collaborating with rehabilitation coordinators had the strongest adjusted odds ratio (OR) for participating in stakeholder meetings, OR 2.72 (95% confidence interval (CI) 2.24–3.31), and having other contacts with employers, OR 3.85 (95% CI 2.85–5.21). Other factors positively associated with employer contacts were being a specialist, collaborating with other health professionals, finding employer contacts valuable, and having a joint SC routine/policy at the clinic. GPs who did not find SC problematic, had managerial support, or had enough resources for SC tasks were more likely to be satisfied with their employer contacts. ***Conclusions:* Both physician characteristics and organizational factors had importance for GPs’ contacts with sick-listed patients’ employers. The findings imply that GPs’ collaboration with patients’ employers may be improved by interventions targeting both individual and organizational factors.**

## Background

High sick-leave rates are a concern for society as it is burdensome for individuals, employers, healthcare, and social insurance systems. In many countries, various interventions have been made to improve the sickness certification (SC) process [1–5]. In Sweden, as in other countries, there has been a strong focus on collaboration and coordination of stakeholders, in particular to involve the employer in the process [6–9]. Collaboration between stakeholders such as healthcare professionals, patients, and employers is considered important for a successful return to work (RTW) process for sick-listed patients [4,10].

In Sweden, all individuals with income from work, unemployment benefits, or parental leave benefits can receive sick-leave benefits if their work capacity is reduced due to injury or disease. From the eighth day of a sick-leave spell, a sickness certificate issued by a physician is needed. The certificate should include information about diagnosis, reduced functioning due to the diagnosis, and the subsequent effects on the patient’s work capacity in relation to her/his work demands. This information constitutes the decision basis for whether the patient is entitled to sick-leave benefits or not.

Research shows that physicians in general [11,12], and general practitioners (GPs) in particular [13,14], find SC problematic, especially to assess patients’ work capacity [11]. According to established models, work capacity is the result of a dynamic and complex interplay between the resources of the individual and the demands of the environment [15–17]. Thus, the assessment of work capacity requires knowledge of (at least) the individual’s level of functioning, his/her work demands, and workplace conditions. This information is also valuable when planning treatment and rehabilitation measures. However, physicians’ knowledge of specific workplaces is limited; their main source of information is usually only the patient while contacts with employers are rare [18].

Qualitative studies have identified different barriers to collaboration between GPs and patient’s employers: organizational factors such as lack of time and channels for communication [19–21], ethical/legal aspects such as confidentiality [2,19,21], stakeholders’ attitudes towards collaboration [19,22], and different perspectives and goals regarding RTW [20,21]. However, there is a lack of quantitative studies and results based on larger samples. The aim of this study was to explore GPs’ contacts with sick-listed patients’ employers and possible associations of such contacts with GP characteristics and organizational factors at the primary healthcare unit.

## Methods

### Study population and design

A cross-sectional study was conducted, based on data from a comprehensive questionnaire sent to all physicians in clinics handling SC of patients, ⩽68 years of age, who worked and lived in Sweden in 2017 (*n* = 34,585). The questionnaire contained 133 questions regarding physicians’ SC practices [23] and was administered by Statistics Sweden. It was sent to the physicians’ home addresses, with up to four reminders to non-responders. The response rate was 54.1% (*n* = 18,714) in total, somewhat higher among women and older physicians.

Our study population consisted of the 4514 responders who stated mainly working within primary healthcare (hereafter referred to as GPs), had been working clinically for the last 12 months, and had SC consultations at least a few times a year. GPs who answered ‘yes’ or did not respond to the question about whether they were working mainly as a locum (defined as working less than one month at each workplace) were excluded (*n* = 286). The final study population consisted of 4228 GPs.

From the questionnaire, answers to 20 questions concerning physician-related or organizational factors were dichotomized and analyzed as independent variables. Answers to the following three questions concerning the frequency and quality of physicians’ contacts with employers were analyzed as dependent variables:

1) *How often in your clinical work do you/your healthcare team participate in stakeholder meetings regarding sickness certified patients?* with seven answer options, dichotomized to ‘Participate in stakeholder meetings’ (>10 times/week, 6–10 times/week, 1–5 times/week, about once a month) vs ‘Not’ (a few times a year, never or almost never).
  Stakeholder meetings are initiated by the Swedish Social Insurance Agency in some SC cases and generally include patient, social insurance officer, employer or employment officer, and treating physician or other healthcare professional from the healthcare team. Employers are not always present at these meetings, yet participating in stakeholder meetings is one arena where contacts between GP and employer may take place.
2) *How often in your clinical work do you/your healthcare team have contacts with patients’ employers in other ways than through stakeholder meetings?* with answer options dichotomized to ‘Have other contacts with employers’ (>10 times/week, 6–10 times/week, 1–5 times/week, about once a month) vs ‘Not’ (a few times a year, never or almost never). These contacts could, for example, be initiated by the GP or the patient.
3) *How satisfied are you with your contacts with patients’ employers?* with four answer options dichotomized to ‘Satisfied’ (very, fairly) vs ‘Not satisfied’ (somewhat, not at all). When analyzing this outcome, the GPs reporting that they did not have any employer contacts were excluded.

All included questions, their response alternatives, and used dichotomizations are presented in Table I. In our sample, missing responses ranged between 0 and 6.6% (mean 1.8%) for included questions; no pattern was identified.

**Table I.** Included questions, their response options, and the dichotomized variables used in the analyses. Questions in bold are outcome variables.

Complete questions	Response options	Dichotomization
***How often in your clinical work . . .***		
**. . . do you/your healthcare team participate in stakeholder meetings regarding sick-listed patients?**	>10 times a week; 6–10 times a week; 1–5 times a week; about once a month; a few times a year; never or almost never	Participate in stakeholder meetings regularly (>10 times a week/6–10 times a week/1–5 times a week/about once a month) vs Not (a few times a year/never or almost never)
**. . . do you/your healthcare team have contacts with employers in other ways than through stakeholder meetings?**	>10 times a week; 6–10 times a week; 1–5 times a week; about once a month; a few times a year; never or almost never	Have other contacts with employers regularly (>10 times a week/6–10 times a week/1–5 times a week/about once a month) vs Not (a few times a year/never or almost never)
**How satisfied are you with your contacts with patients’ employers?**	Very; fairly; somewhat; not at all; have no contacts with patients’ employers*	Satisfied (very/fairly) vs Not satisfied (somewhat/not at all)
How problematic do you in general find it to handle SC of patients?	Very; somewhat; a little; not at all	Problematic (very/somewhat) vs Not problematic (not so much/not at all)
*How do you value the following alternatives with regard to ensuring high quality in your handling of SC, now and in the future?*		
Stakeholder meetings	Great value; moderate value; no value	Valuable (great value/moderate value) vs Not valuable (no value)
Contacts with patients’ employers	Great value; moderate value; no value	Valuable (great value/moderate value) vs Not valuable (no value)
*To what extent do you agree with the statements below?*		
How I handle SC is important for my patients	Totally agree; agree to a large extent; hardly agree; do not agree at all	Agree (totally agree/agree to a large extent) vs Do not agree (hardly agree/do not agree at all)
Focus on return to work is valuable in my work with SC	Totally agree; agree to a large extent; hardly agree; do not agree at all	Agree (totally agree/agree to a large extent) vs Do not agree (hardly agree/do not agree at all)
I have sufficient resources to do a good job with SC cases	Totally agree; agree to a large extent; hardly agree; do not agree at all	Agree (totally agree/agree to a large extent) vs Do not agree (hardly agree/do not agree at all)
*How often in your clinical work do you. . .*		
. . . experience that your competence in insurance medicine is not sufficient?	>10 times a week; 6–10 times a week; 1–5 times a week; about once a month; a few times a year; never or almost never	Regularly (>10 times a week/6–10 times a week/1–5 times a week/about once a month) vs Seldom or never (a few times a year/never or almost never)
. . . confer with other physicians in SC cases?	>10 times a week; 6–10 times a week; 1–5 times a week; about once a month; a few times a year; never or almost never	Regularly (>10 times a week/6–10 times a week/1–5 times a week/about once a month) vs Seldom or never (a few times a year/never or almost never)
. . . collaborate with or refer patients to a rehabilitation coordinator in SC cases?	>10 times a week; 6–10 times a week; 1–5 times a week; about once a month; a few times a year; never or almost never	Regularly (>10 times a week/6–10 times a week/1–5 times a week/about once a month) vs Seldom or never (a few times a year/never or almost never)
. . . collaborate with or refer patients to a medical social worker or psychologist in SC cases?	>10 times a week; 6–10 times a week; 1–5 times a week; about once a month; a few times a year; never or almost never	Regularly (>10 times a week/6–10 times a week/1–5 times a week/about once a month) vs Seldom or never (a few times a year/never or almost never)
. . . collaborate with or refer patients to physical or occupational therapists in SC cases?	>10 times a week; 6–10 times a week; 1–5 times a week; about once a month; a few times a year; never or almost never	Regularly (>10 times a week/6–10 times a week/1–5 times a week/about once a month) vs Seldom or never (a few times a year/never or almost never)
. . . refer/send patients to occupational health services?	>10 times a week; 6–10 times a week; 1–5 times a week; about once a month; a few times a year; never or almost never	Regularly (>10 times a week/6–10 times a week/1–5 times a week/about once a month) vs Seldom or never (a few times a year/never or almost never)
Does your clinical unit have routines/a joint policy for handling SC cases?	Yes and it is well established; yes but it is not well established; no; I don’t know	Yes (yes and it is well established/yes but it is not well established) vs No (no/I don’t know)
If so, is the policy useful to you in your clinical work?	Yes; no	Yes vs No
When handling SC cases, how often do you not have enough time to manage patient-related aspects (e.g., issuing certificates, contacting other stakeholders, documentation, and meetings)?	Every day; about once a week; about once a month; a few times a year; never or almost never	At least once a week (every day/about once a week) vs Once a month or less (about once a month/a few times a year/never or almost never)
Do you have access to a so-called rehabilitation coordinator at your clinical unit?	Yes; no; I don’t know	Yes vs No (no/I don’t know)
Do you have support from your immediate manager at your clinic regarding handling SC cases?	Substantial support; some support; no; not applicable	Yes (substantial support/some support vs No (no/not applicable)

* Individuals choosing this response option were excluded from the analysis of this question.

SC: sickness certification.

### Data analyses

Descriptive statistics were used to describe the characteristics of the study population. Bivariate and multiple logistic regressions were performed to explore associations between independent variables and outcomes, calculated as crude and adjusted odds ratios (OR) with 95% confidence intervals (CI). In the first multivariate analyses, independent variables were grouped into two sets – physician-related and organizational – and the sets were analyzed separately, with all variables within each set entered regardless of crude estimates. In the final analysis (full model), all variables from both sets were entered. Multicollinearity among independent variables was checked for using Spearman’s correlation coefficient (limit *r* > 0.70); none was found. Analyses were performed using IBM SPSS Statistics Version 26.0 (IBM Corp., Armonk, NY).

The project was approved by the Regional Ethical Review Board of Stockholm, Sweden.

## Results

Among the 4228 GPs, 60.2% were certified specialists and 41.5% had been at the same primary healthcare center, hereafter called clinic, for at least five years (Table II). Slightly higher rates of the GPs were women and younger.

**Table II.** Characteristics of the 4228 general practitioners included in the study.

Variable	*n* (%)
*Physician-related factors*	
Woman	2390 (56.5)
24–39 years	1601 (37.9)
40–54 years	1448 (34.2)
55–68 years	1179 (27.9)
Certified specialist	2542 (60.2)
Worked ⩾5 years at current workplace	1748 (41.5)
Experience own insurance medical competence as insufficient regularly^a^	2229 (53.6)
Find SC problematic	3211 (77.2)
Find contacts with employers valuable	3520 (86.4)
Find stakeholder meetings valuable	3854 (94.6)
Agree that one’s SC practices are important for the patients	3990 (96.1)
Agree that focus on return to work is valuable in one’s work with SC	3955 (95.7)
Consult other physicians regularly^a^ in SC cases	1937 (46.3)
Cooperate with rehabilitation coordinator regularly^a^	1914 (46.0)
Cooperate with medical social worker or psychologist regularly^a^ in SC cases	3147 (75.4)
Cooperate with physiotherapist or occupational therapist regularly^a^ in SC cases	3123 (75.1)
Refer patients to occupational health services regularly^a^	2193 (52.7)
*Organizational factors*	
Have joint SC routines/policy at the primary healthcare center	2198 (52.2)
Benefit from the SC routines/policy (among those who have such)	1559 (75.9)
Have lack of time for patient-related SC tasks (such as contacts with other stakeholders) at least once a week	3544 (85.3)
Have access to rehabilitation coordinator	2791 (66.2)
Have support from management in SC matters	3295 (79.3)
Have enough resources to do a good job with SC	1546 (37.3)
*Outcomes*	
Participate in stakeholder meetings regularly^a^	1447 (34.8)
Have other contacts with employers regularly^a^	627 (15.1)
Satisfied with employer contacts^b^	1481 (65.8)

SC: sickness certification.

a At least once a month.

b Among those who reported having contacts with employers in this specific question.

### Participation in stakeholder meetings

A total of 34.8% of GPs reported participating in stakeholder meetings (Table II). Certified specialists were twice as likely (OR 1.97; 95% CI 1.64–2.38) to participate in such meetings as those not yet specialists (Table III). The factor with the highest OR for participating in stakeholder meetings was regular collaboration with rehabilitation coordinator (OR 2.72; 95% CI 2.24–3.31). Having other types of collaboration within the clinic also showed a positive association with participating in stakeholder meetings, but ORs were lower. The organizational factor most strongly associated with participation in stakeholder meetings in adjusted analyses was having lack of time for patient-related SC tasks at least once a week.

**Table III.** Factors associated with general practitioners and/or their healthcare team participating in stakeholder meetings regularly or not. Odds ratios (OR) with corresponding 95% confidence intervals (CI) for crude model, model with sets of variables, and full model, respectively. Numbers in bold indicate a statistically significant association (*p* < 0.05).

Variables, dichotomized	Participate in stakeholder meetings regularly^a^		
	Logistic regression, crude	Logistic regression, sets of variables^b^	Logistic regression, full model
	OR (95% CI)	OR (95% CI)	OR (95% CI)
*Physician-related factors*			
Woman	**0.83** (0.73–0.94)	**0.83** (0.71–0.96)	**0.83** (0.71–0.96)
Certified specialist	**1.79** (1.56–2.04)	**1.99** (1.66–2.39)	**1.97** (1.64–2.38)
Worked ⩾5 years at current workplace	**1.52** (1.33–1.73)	**1.19** (1.01–1.41)	1.15 (0.97–1.37)
Experience own insurance medical competence as insufficient regularly^a^	1.06 (0.93–1.20)	1.00 (0.86–1.17)	1.00 (0.85–1.18)
Find SC problematic	1.07 (0.92–1.25)	1.05 (0.88–1.27)	1.03 (0.85–1.25)
Find contacts with employers valuable	**1.44** (1.18–1.75)	1.08 (0.85–1.37)	1.10 (0.86–1.41)
Find stakeholder meetings valuable	**2.71** (1.89–3.89)	**2.34** (1.52–3.59)	**2.13** (1.38–3.30)
Agree that one’s SC practices are important for the patients	0.80 (0.58–1.10)	0.68 (0.46–1.01)	**0.64** (0.43–0.94)
Agree that focus on return to work is valuable in one’s work with SC	0.74 (0.55–1.01)	0.70 (0.49–1.02)	0.69 (0.47–1.00)
Consult other physicians regularly^a^ in SC cases	**1.66** (1.46–1.89)	**1.64** (1.40–1.92)	**1.59** (1.35–1.87)
Cooperate with rehabilitation coordinator regularly^a^	**4.67** (4.07–5.37)	**3.45** (2.96–4.03)	**2.72** (2.24–3.31)
Cooperate with medical social worker or psychologist regularly^a^ in SC cases	**3.37** (2.82–4.03)	**1.51** (1.19–1.91)	**1.47** (1.15–1.86)
Cooperate with physiotherapist or occupational therapist regularly^a^ in SC cases	**3.17** (2.66–3.77)	**1.57** (1.25–1.98)	**1.54** (1.22–1.95)
Refer patients to occupational health services regularly^a^	**1.35** (1.19–1.54)	**1.25** (1.07–1.45)	**1.22** (1.05–1.42)
*Organizational factors*			
Have joint SC routines/policy at the primary healthcare center	**1.99** (1.75–2.27)	**1.58** (1.38–1.83)	**1.18** (1.01–1.39)
Benefit from the SC routines/policy^c^	**1.65** (1.33–2.04)	–	–
Have lack of time for patient-related SC tasks at least once a week	**1.86** (1.52–2.27)	**2.04** (1.65–2.53)	**1.60** (1.26–2.03)
Have access to rehabilitation coordinator	**3.42** (2.93–3.99)	**3.02** (2.56–3.55)	**1.41** (1.13–1.76)
Have support from management in SC matters	**1.58** (1.33–1.86)	**1.27** (1.06–1.52)	1.17 (0.95–1.43)
Have enough resources to do a good job with SC	1.04 (0.91–1.19)	1.05 (0.91–1.22)	1.05 (0.89–1.24)

SC: sickness certification.

a At least once a month.

b Physician-related factors and organizational factors analyzed within their respective set.

c Analyzed only among those who reported having joint routines/policy, and only in crude analysis.

### Other contacts with employers

Having other types of contacts with employers in SC cases was reported by 15.1% of the GPs (Table II). GPs who reported regularly collaborating with a rehabilitation coordinator were almost four times as likely (OR 3.85; 95% CI 2.85–5.21) to have other contacts with employers as those who did not (Table IV). Those who regularly had other types of collaborations within the clinic or had a joint SC routine/policy at the clinic also had higher odds of having other contacts with employers.

**Table IV.** Factors associated with general practitioners and/or their healthcare team regularly having contacts with employers in other ways than through stakeholder meetings. Odds ratios (OR) with corresponding 95% confidence intervals (CI) for crude model, model with sets of variables, and full model, respectively. Numbers in bold indicate a statistically significant association (*p* < 0.05).

Variables, dichotomized	Have other contacts with employers regularly^a^		
	Logistic regression, crude	Logistic regression, sets of variables^b^	Logistic regression, full model
	OR (95% CI)	OR (95% CI)	OR (95% CI)
*Physician-related factors*			
Woman	0.89 (0.75–1.05)	0.84 (0.69–1.01)	0.85 (0.70–1.03)
Certified specialist	**1.35** (1.13–1.62)	**1.55** (1.23–1.96)	**1.52** (1.19–1.93)
Worked ⩾5 years at current workplace	**1.21** (1.02–1.44)	1.00 (0.80–1.25)	0.98 (0.78–1.22)
Experience own insurance medical competence as insufficient regularly^a^	1.18 (0.99–1.40)	1.09 (0.89–1.34)	1.15 (0.93–1.42)
Find SC problematic	0.87 (0.72–1.07)	**0.79** (0.63–0.99)	0.82 (0.64–1.05)
Find contacts with employers valuable	**2.21** (1.60–3.04)	**1.72** (1.19–2.49)	**1.80** (1.24–2.63)
Find stakeholder meetings valuable	**2.65** (1.53–4.59)	1.67 (0.87–3.14)	1.56 (0.81–2.98)
Agree that one’s SC practices are important for the patients	0.89 (0.58–1.37)	0.71 (0.43–1.17)	0.70 (0.42–1.16)
Agree that focus on return to work is valuable in one’s work with SC	0.88 (0.59–1.33)	0.77 (0.49–1.23)	0.74 (0.46–1.19)
Consult other physicians regularly^a^ in SC cases	**2.19** (1.84–2.61)	**1.81** (1.48–2.23)	**1.76** (1.43–2.17)
Cooperate with rehabilitation coordinator regularly^a^	**6.12** (4.97–7.53)	**4.45** (3.54–5.59)	**3.85** (2.85–5.21)
Cooperate with medical social worker or psychologist regularly^a^ in SC cases	**3.89** (2.92–5.19)	1.40 (0.98–2.01)	1.39 (0.96–2.00)
Cooperate with physiotherapist or occupational therapist regularly^a^ in SC cases	**3.74** (2.83–4.95)	**1.65** (1.17–2.33)	**1.54** (1.09–2.18)
Refer patients to occupational health services regularly^a^	**1.78** (1.49–2.12)	**1.63** (1.34–1.99)	**1.63** (1.33–2.00)
*Organizational factors*			
Have joint SC routines/policy at the primary healthcare center	**2.50** (2.08–3.01)	**1.94** (1.60–2.35)	**1.54** (1.24–1.90)
Benefit from the SC routines/policy^c^	**2.11** (1.57–2.82)	–	–
Have lack of time for patient-related SC tasks at least once a week	**1.43** (1.09–1.86)	**1.58** (1.19–2.10)	1.20 (0.87–1.64)
Have access to rehabilitation coordinator	**3.94** (3.10–5.02)	**3.31** (2.57–4.26)	1.14 (0.80–1.61)
Have support from management in SC matters	**1.74** (1.37–2.22)	1.27 (0.99–1.64)	0.99 (0.75–1.31)
Have enough resources to do a good job with SC	**1.25** (1.05–1.49)	**1.21** (1.01–1.46)	1.23 (1.00–1.52)

SC: sickness certification.

a At least once a month.

b Physician-related factors and organizational factors analyzed within their respective set.

c Analyzed only among those who reported having joint routines/policy, and only in crude analysis.

In all, 39.4% of the GPs had any or both of these two types of employer contacts in SC cases.

### Satisfaction with employer contacts

In the question about satisfaction with employer contacts, 2250 GPs (53.2% of all) reported having contacts with sick-listed patients’ employers and of them, 65.8% reported being satisfied with those contacts (Table II). The most important factor for being satisfied was agreeing that one’s SC practices are important for the patient (OR 3.64; 95% CI 2.05–6.47) (Table V). GPs who found SC problematic had lower odds of being satisfied (OR 0.53; 95% CI 0.41–0.69), while the odds for being satisfied was higher among GPs who stated having enough resources for SC matters.

**Table V.** Factors associated with general practitioners (*n* = 2250) being satisfied or not with their contacts with sick-listed patients’ employers. Odds ratios (OR) with corresponding 95% confidence intervals (CI) for crude model, model with sets of variables, and full model, respectively. Numbers in bold indicate a statistically significant association (*p* < 0.05).

Variables, dichotomized	Satisfied with contacts		
	Logistic regression, crude	Logistic regression, sets of variables^b^	Logistic regression, full model
	OR (95% CI)	OR (95% CI)	OR (95% CI)
*Physician-related factors*			
Woman	**1.24** (1.04–1.48)	**1.26** (1.05–1.52)	**1.27** (1.05–1.54)
Certified specialist	1.05 (0.87–1.26)	0.96 (0.76–1.20)	1.00 (0.79–1.26)
Worked ⩾5 years at current workplace	1.17 (0.98–1.40)	1.11 (0.90–1.37)	1.12 (0.90–1.40)
Experience own insurance medical competence as insufficient regularly^a^	**0.68** (0.57–0.81)	**0.79** (0.65–0.96)	0.84 (0.68–1.03)
Find SC problematic	**0.45** (0.36–0.56)	**0.48** (0.38–0.62)	**0.53** (0.41–0.69)
Find contacts with employers valuable	**1.48** (1.06–2.07)	1.33 (0.91–1.94)	1.33 (0.90–1.96)
Find stakeholder meetings valuable	1.24 (0.74–2.08)	1.02 (0.57–1.83)	1.05 (0.58–1.90)
Agree that one’s SC practices are important for the patients	**4.67** (2.74–7.96)	**3.90** (2.20–6.90)	**3.64** (2.05–6.47)
Agree that focus on return to work is valuable in one’s work with SC	0.99 (0.62–1.58)	0.70 (0.42–1.16)	0.61 (0.36–1.03)
Consult other physicians regularly^a^ in SC cases	1.03 (0.87–1.23)	1.02 (0.84–1.25)	0.96 (0.78–1.18)
Cooperate with rehabilitation coordinator regularly^a^	**1.34** (1.12–1.59)	**1.33** (1.08–1.62)	**1.38** (1.08–1.77)
Cooperate with medical social worker or psychologist regularly^a^ in SC cases	1.08 (0.87–1.33)	1.01 (0.76–1.36)	1.03 (0.77–1.38)
Cooperate with physiotherapist or occupational therapist regularly^a^ in SC cases	1.04 (0.84–1.29)	0.96 (0.72–1.27)	0.96 (0.72–1.29)
Refer patients to occupational health services regularly^a^	**0.82** (0.69–0.98)	0.85 (0.70–1.03)	0.85 (0.70–1.03)
*Organizational factors*			
Have joint SC routines/policy at the primary healthcare center	1.14 (0.96–1.37)	0.99 (0.82–1.20)	0.93 (0.76–1.15)
Benefit from the routines/policy^c^	**1.42** (1.08–1.87)	–	–
Have lack of time for patient-related SC tasks at least once a week	**0.72** (0.54–0.95)	0.92 (0.69–1.23)	1.01 (0.74–1.39)
Have access to rehabilitation coordinator	1.17 (0.97–1.42)	1.11 (0.91–1.37)	0.89 (0.69–1.17)
Have support from management in SC cases	**1.46** (1.17–1.83)	**1.35** (1.07–1.71)	**1.34** (1.04–1.73)
Have enough resources to do a good job with SC	**1.81** (1.50–2.19)	**1.77** (1.45–2.16)	**1.45** (1.16–1.80)

SC: sickness certification.

a At least once a month.

b Physician-related factors and organizational factors analyzed within their respective set.

c Analyzed only among those who reported having joint routines/policy, and only in crude analysis.

## Discussion

In this large nationwide survey, four out of 10 GPs reported participating in stakeholder meetings and/or having other contacts with employers regarding their sick-listed patients. A majority of those who had employer contacts were satisfied with these contacts. Being a certified specialist, finding employer contacts valuable, collaborating with other health professionals, in particular rehabilitation coordinators, and having a joint SC routine/policy at the clinic were factors positively associated with having contacts with employers. GPs who stated that they had managerial support and sufficient resources in their work with SC were more likely to report being satisfied with their employer contacts.

### Participation in stakeholder meetings

GPs having access to or cooperating with a rehabilitation coordinator were more likely to participate in stakeholder meetings. The rehabilitation coordinator is a relatively new function in Swedish healthcare, since 2020 mandatory by law, and handles internal coordination, external contacts, and individual support to sick-listed patients. Stakeholder meetings are now often initiated by the rehabilitation coordinator, which could explain the strong associations.

In fact, collaboration with all types of healthcare professionals was positively associated with participation in stakeholder meetings. This could indicate that a general collaborative approach within the clinic also supports collaboration outside the clinic. In their review of collaboration in vocational rehabilitation, Andersson et al. found that an important facilitator for collaboration is that it is promoted and supported by the management [24].

The finding that lack of time for patient-related SC tasks was positively associated with participation in stakeholder meetings was unexpected. In previous studies, lack of time has been lifted as a barrier for collaboration [19,20,24]. Since stakeholder meetings take time, they could be contributing to time pressure for GPs who participate in them. Another interpretation could be that GPs who experience a lack of time in their SC work consider such meetings efficient [25], and, therefore, participate (in person or by delegating to, e.g., rehabilitation coordinators) to a higher degree than other GPs.

### Other contacts with employers

Cooperating with rehabilitation coordinator or other health professionals were factors positively associated with having other types of contacts with employers. This highlights that a collaborative environment also goes with more frequent contacts with employers other than through stakeholder meetings. GPs reporting having a joint SC routine/policy at the clinic were also more likely to have other contacts with employers. This is in line with previous research on collaboration in the SC process, stating that organizational resources are significant facilitators for collaboration [7,24].

### Satisfaction with employer contacts

GPs who found SC problematic were less likely to be satisfied with their employer contacts. Suboptimal employer contacts, in terms of format (indirect rather than direct contacts), content (employers are not cooperating, do not reach solutions etc.), and/or frequency (not enough contacts), could add to experiencing this as problematic [26]. In contrast, those experiencing managerial support and having enough resources in their work with SC had higher odds for being satisfied. This is consistent with the previously mentioned findings about the importance of a supportive management and explicit organizational resources [7,24].

### GP characteristics and organizational factors

Our findings suggest that both physician-related and organizational factors are important for GPs’ contacts with sick-listed patient’s employers. Cooperation with rehabilitation coordinator was the factor that remained positively associated with all outcomes throughout our analyses. Here, cooperation was defined as a physician characteristic, referring to a collaborative behavior. Ståhl et al. [22] showed that GPs’ attitudes to collaboration can vary and influence whether collaboration takes place or not. In line with this, we found that GPs who found stakeholder meetings or other employer contacts valuable were more likely to have such contacts. However, cooperation is also closely related to organizational resources and routines. For example, the rehabilitation coordinator can be employed full-time or on a very limited part-time – if more available, cooperation is facilitated, for example, if the rehabilitation coordinator is at the clinic when the GP has a patient to discuss. Such opportunities have been described by GPs as a valuable ‘informal support’ in their work with SC [25]. Trustful relationships between healthcare professionals at the clinic, including rehabilitation coordinators and GPs, is another factor of importance [7]. GPs sometimes describe themselves as the patient’s advocate in SC [19], and to involve other professionals might not be easy if trust is failing. Finally, as discussed above, supportive management at the clinic is important to lift SC issues and make room for professional handling of these tasks. In addition, it is quite possible that the broad spectrum of diagnoses in primary healthcare, and the high prevalence of mental disorders, which are considered more difficult in relation to SC [27,28], make the need for explicit organizational support even greater in primary healthcare than in other types of clinics.

#### Methodological considerations

The study has several strengths: the questionnaire and methods for data collection have been developed and refined during 15 years to adequately examine the area of SC, all GPs working and living in Sweden were invited, the response rate was acceptable, and the number of included physicians was large, allowing for subgroup analyses. We do not know how non-responders would have reported on the subject being studied. The study also has some important limitations, one being that two of the outcome questions do not measure only GPs’ contacts with employers, the answers also include such contacts delegated to other healthcare professionals in the team. Second, as in all questionnaire studies, we cannot be sure how the participants interpreted the questions. Third, all organizational factors were self-reported by the GPs. For example, all healthcare centers are expected to have joint SC routines/policies; however, the focus here was whether the individual GP actually knew about them. Also, recall bias regarding frequencies may occur; however, is not expected to differ between the groups.

## Conclusions

Both physician-related and organizational factors were of importance for GPs having and being satisfied with contacts with sick-listed patients’ employers. The findings imply that GPs’ collaboration with patients’ employers may be improved by interventions targeting both individual and organizational factors.

## Supplemental Material

sj-doc-1-sjp-10.1177_14034948211053141 – Supplemental material for Factors associated with general practitioners’ contacts with sick-listed patients’ employers: A Swedish nationwide questionnaire study Click here for additional data file. Supplemental material, sj-doc-1-sjp-10.1177_14034948211053141 for Factors associated with general practitioners’ contacts with sick-listed patients’ employers: A Swedish nationwide questionnaire study by Paula Nordling, Kristina Alexanderson, Gunnel Hensing and Per Lytsy in Scandinavian Journal of Public Health
