# Supplementary material for: Factors associated with general practitioners’ contacts with sick-listed patients’ employers: A Swedish nationwide questionnaire study
Source: Scand J Public Health. 2021 Oct 23;51(4):602–10. doi: 10.1177/14034948211053141 (PMC10854204; doi:10.1177/14034948211053141)
Supplement: sj-doc-1-sjp-10.1177_14034948211053141 – Supplemental material for Factors associated with general practitioners’ contacts with sick-listed patients’ employers: A Swedish nationwide questionnaire study [file sj-doc-1-sjp-10.1177_14034948211053141.doc]

All physicians in clinics handling SC of patients, <68 years of age, working and living in Sweden in 2017 (n=34,585)

Non-responders (n=15,871)

Responders (n=18,714)

Excluded (n=14,200)

- Did not handle SC or had not worked clinically during the last 12 months (n=4964)
- Worked in privately financed healthcare (n=675)
- Did not work in primary healthcare (n=8561)

Study population (n=4514)

Excluded (n=286)

- Worked mainly as a locum (n=286)

Final study population (n=4228)

Supplementary Figure 1. Flow chart presenting the selection process for the study population.
